# Short-Term Changes in Light Distortion in Orthokeratology Subjects

**DOI:** 10.1155/2015/278425

**Published:** 2015-01-28

**Authors:** Elena Santolaria Sanz, Alejandro Cerviño, Antonio Queiros, Cesar Villa-Collar, Daniela Lopes-Ferreira, Jose Manuel González-Méijome

**Affiliations:** ^1^Private Practice, Onda, 12200 Castellon, Spain; ^2^Optometry Research Group, Department of Optics, Universidad de Valencia, 46100 Valencia, Spain; ^3^Clinical & Experimental Optometry Research Lab (CEORLab), Center of Physics (Optometry), Universidade do Minho, 4710-057 Braga, Portugal; ^4^Department of Optics and Optometry, Universidad Europea de Madrid, 28670 Villaviciosa de Odón, Spain

## Abstract

*Purpose*. Quantifying adaptation to light distortion of subjects undergoing orthokeratology (OK) for myopia during the first month of treatment.* Methods*. Twenty-nine healthy volunteers (age: 22.34 ± 8.08 years) with mean spherical equivalent refractive error −2.10 ± 0.93D were evaluated at baseline and days 1, 7, 15, and 30 of OK treatment. Light distortion was determined using an experimental prototype. Corneal aberrations were derived from corneal topography for different pupil sizes. Contrast sensitivity function (CSF) was analyzed for frequencies of 1.50, 2.12, 3.00, 4.24, 6.00, 8.49, 12.00, 16.97, and 24.00 cpd under photopic conditions.* Results*. Average monocular values of all light distortion parameters measured increased significantly on day 1, returning to baseline after 1 week (*P* < 0.05 in all cases). Spherical-like aberration stabilized on day 7 for all pupil diameters, while coma-like for smaller pupils only. CSF was significantly reduced on day 1 for all spatial frequencies except for 1.5 cpd, returning to baseline afterwards. Significant correlation was found between light distortion and contrast sensitivity for middle and high frequencies (*P* < 0.05) after 15 days.* Conclusion*. Despite consistently increased levels of corneal aberrations, light distortion tends to return to baseline after one week of treatment, suggesting that neural adaptation is capable of overcoming optical quality degradation.

## 1. Introduction

Modern corneal refractive therapy (CRT) or orthokeratology (OK) aims to reshape the anterior corneal surface by the overnight application of reverse geometry contact lenses (CL). In the case of myopia correction, the central cornea is flattened to achieve the desired reduction in the power of the anterior corneal surface, while the midperipheral cornea steepens [1, 2] as a result of the epithelial thickness redistribution from the corneal center. These histological modifications occurring in orthokeratology [3, 4] drive a change in the optical quality of the cornea, significantly increasing spherical aberration in the positive direction [5, 6] with an impact on visual quality, particularly under low luminance conditions [7–9].

In clinical practice, OK subjects used to complain of dysphotopic phenomena in the form of haloes, ghosting, or glare. These phenomena are described as more intense at the beginning of the treatment period and decrease over time [8, 10] and might also be associated with a loss in visual quality, but none of them has been analyzed further yet. Indeed, it is assumed that the increase in corneal higher order aberrations and related optical quality degradation will remain during the treatment [11]; thus, we hypothesize that the increase in light distortion phenomena is a transient process that might be independent of the optical quality changes in the corneal surface and might have an impact on visual quality.

The purpose of this study was to evaluate the time-course variations in the size of light distortion phenomena measured with an experimental device and correlate them with the aberrations of the anterior corneal surface and contrast sensitivity function (CSF) over the first month of treatment.

## 2. Methods

A total of 29 neophyte subjects were recruited and fitted with OK lenses for myopia correction with reverse geometry rigid gas permeable CL. Inclusion criteria required that they be over 18 years of age, have less than 1.00 diopters (D) of refractive astigmatism, be free of ocular disease, have no contraindication for overnight CL wear, and present a best spectacle corrected monocular visual acuity of 0.90 decimal (20/25) or better. Subjects were followed up and they wore their lenses successfully for 1 month. Demographic and refractive data of subjects are presented in Table 1. All subjects enrolled achieved a satisfactory correction and no dropouts were observed during the 1-month follow-up time.

Subjects were informed of the purpose of the study and signed a consent form after all their questions were answered following the tenets of the Declaration of Helsinki. Subjects underwent a comprehensive optometric examination prior to enrollment.

### 2.1. Outcome Measures

Visual acuity was measured under photopic conditions at 5 m using a decimal scale chart. Subjective baseline refraction and refraction at the time of data collection were determined as the spherocylindrical combination that rendered the best distance visual acuity with the highest positive power.

**Figure 1:**
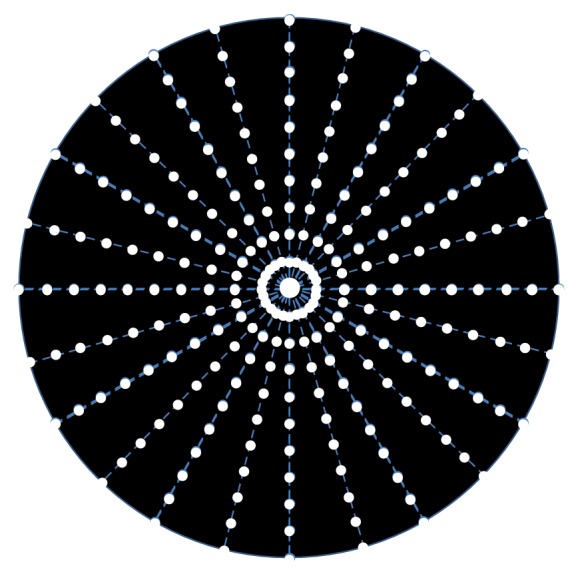
Distribution of main central source of light and peripheral stimuli.

Light distortion was analysed with an experimental prototype consisting of a central light emitting diode (LED) surrounded by 240 small LED sources distributed in 24 semimeridians with an angular separation of 15°. For the purpose of the present experiment, an angular separation of 30° was considered.  Figure 1 represents the arrangement of the central white LEDs and the surrounding smaller white LEDs. The central LED was a commercially available white LED from Agilent Technologies (ref. HLMP-CW47-RU000 from Agilent Technologies, Inc., Berkshire, United Kingdom); the surrounding LEDs were commercially available white LEDs from Avago Technologies (ref. HSMW-CL25 from Avago Technologies, San Jose, California, United States). The subject was at a distance of 2.0 m in a darkened room. The physical (electronic board) display device is connected to a central control device (PC) via USB connection when he/she can detect a peripheral LED as this is moving from the inner part to the outer part of the area of examination. The subject being evaluated provides feedback to the system through a remote response device (PC mouse). Peripheral stimuli are presented around the central source of light from the inner to the outer part of the field at random times ranging from 250 to 750 milliseconds. Semimeridians are explored in random order. When the subject sees the stimulus, he/she presses the mouse control and the system presents the next semimeridian. Further description of the device can be found elsewhere [12]. After data collection and storage, a software tool then calculates three indices that determine the size and regularity of the distortion surrounding the central source of light.

The Best Fit Circle Radius (BFCr) is defined as the circle that best fits the distortion area resulting from the linear binding of all points in each meridian of the device. This parameter is expressed in mm and is linearly related to the Distortion Index (LDI) parameter.

LDI is calculated as the ratio of the area or points missed by the subject and the total area explored and is expressed as a percentage (%). The higher values of distortion (BFCr and LDI) are interpreted as a lower ability to discriminate small stimuli surrounding the central light source.

The irregularity of the distortion area is derived as the deviation of the actual polygonal shape obtained from the BFC fit and is called the BFC Irregularity (BFCirr). The standard deviation of BFCirr, called BFCsd, measures how asymmetric the departure of the actual limits of the distortion from the perfect circular shape of the BFC is. Together, BFCirr and BFCsd can be interpreted as the deviation of the actual distortion from a perfectly rotational symmetric shape. The higher the value of this parameter, the larger the deviation from a circular shape and it is expressed in mm. The device has been applied in several studies to different ocular conditions, showing consistency in its measurements. In a recent study, the system has been able to differentiate the light disturbance between monofocal, bifocal, and trifocal pseudophakic patients (Brito et al., J Cataract Refract Surg,* in press*).

Corneal aberrations were derived from topography data obtained with the Oculus Easygraph (Oculus, Dudenhofen, Germany). Corneal higher order aberrations in the form of spherical-like, coma-like, and secondary astigmatism were calculated from the Zernike coefficients provided by the topographer for 3.0, 4.5, and 6.0 mm pupil sizes.

Measurements of CSF (CSF) were carried out using a 22′′ LCD screen (Topcon CC-100XP 75-1, Hasunuma-cho, Itabashi-ku, Tokyo 174-8580, Japan). The frequencies tested were 1.50, 2.12, 3.00, 4.24, 6.00, 8.49, 12.00, 16.97, and 24.00 cpd. Test distance was 5 m under photopic conditions. This consisted of a total of 81 presentations (9 spatial frequencies at 9 different contrast levels).

Light distortion and CSF parameters were recorded monocularly and binocularly while the subject was wearing the best spherocylindrical overrefraction. For monocular analysis, only the right eye of each subject has been considered. All measurements were obtained at baseline and after 1, 7, 15, and 30 days of treatment.

### 2.2. Statistical Analysis

Statistical analysis was carried out using SPSS software v15.0 (SPSS Inc., Chicago, IL). Descriptive statistics of the variables measured in the study were produced. Normality of data distribution was assessed with Kolmogorov-Smirnov test. All parameters followed a normal distribution (*K*-*S*, *P* > 0.05). Changes in different parameters from baseline to subsequent visits were compared using ANOVA test with Bonferroni correction. Correlations between different parameters were performed with Pearson correlation. Statistical significance criteria were established at *P* < 0.05.

## 3. Results

### 3.1. Light Distortion

**Figure 2:**
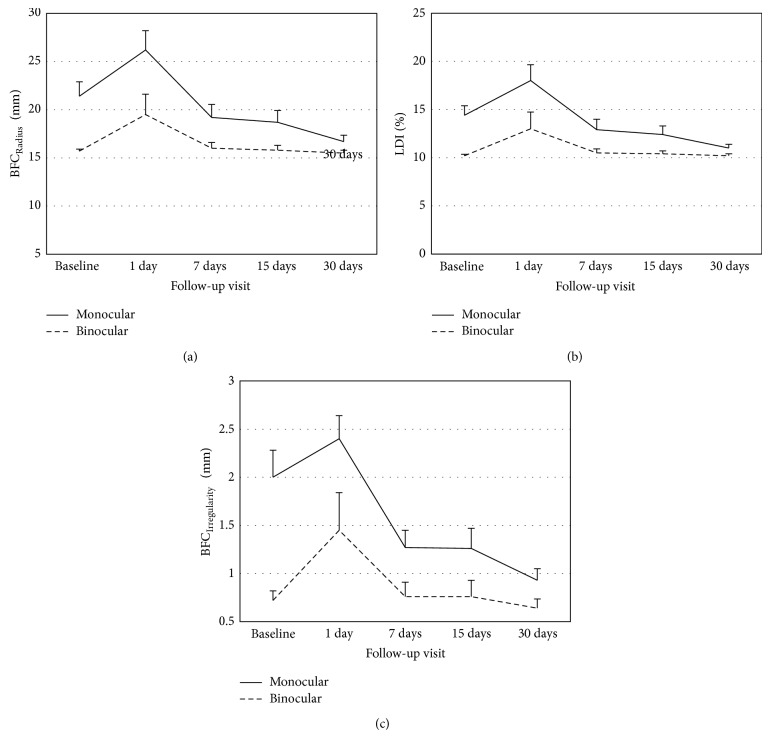
Monocular and binocular Best Fit Circle Radius (BFCr) (a), Light Distortion Index (LDI) (b), and BFC Irregularity (BFC Irregularity) parameter (c) of light distortion. Bars represent the Standard Error of the Mean (SEM).

Size-related light distortion parameters (LDI and BFCr) and regularity-related parameters (BFCirr and BFCsd) showed a statistically significant change over time on ANOVA analysis (*P* < 0.05) but there were no significant changes from baseline to any of the subsequent analyses (Bonferroni correction for multiple comparisons).  Figure 2 exemplifies the variations of size-related and irregularity-related parameters of monocular and binocular light distortion over time. A marked and statistically significant increase on day 1 after treatment onset has been observed.

LDI index returns to baseline on day 7 and remains at baseline level afterwards. Conversely, irregularity parameter (BFCirr) shows a different path displaying a significant reduction after day 7 compared to baseline after a transient increase on day 1. Binocular analysis did not report any statistically significant change from baseline to day 1 or subsequent visits for LDI (*P* > 0.088) or BFCr (*P* > 0.060).

### 3.2. Corneal Aberrations

**Figure 3:**
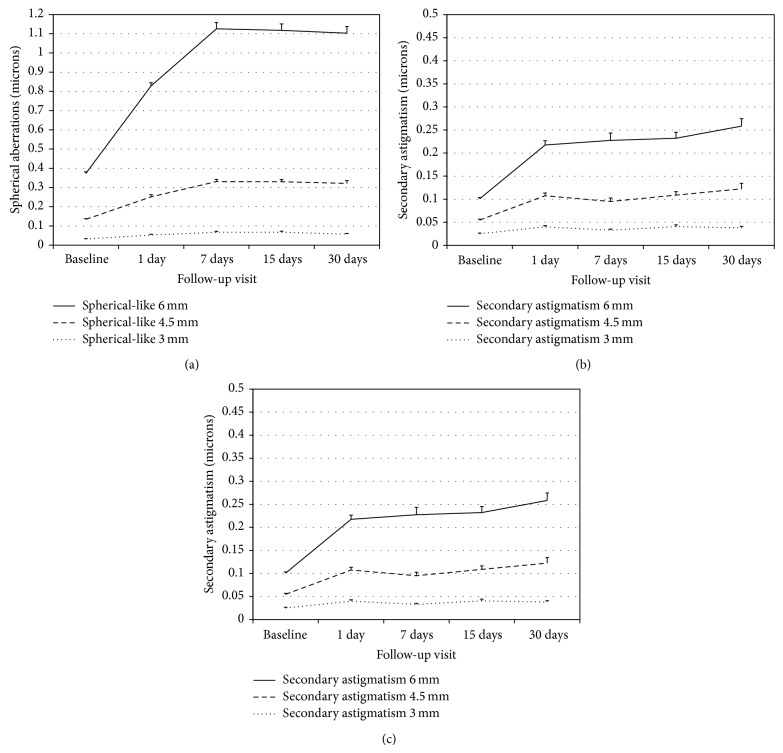
Optical quality of the corneal front surface for different pupil sizes represented by the root mean square (RMS) of spherical-like aberrations (a), coma-like aberrations (b), and secondary astigmatism (c). Bars represent the Standard Error of the Mean (SEM).

There have been statistically significant changes from baseline in all optical quality descriptors of the corneal front surface for the three pupil sizes under analysis (*P* < 0.05, ANOVA with Bonferroni correction), the exception to this being secondary astigmatism for 3.0 mm pupil on day 7 and coma-like aberrations for 6.0 mm pupil on day 1 (*P* > 0.05).  Figure 3 illustrates the changes in corneal front surface optical aberrations for different pupil sizes.

### 3.3. Contrast Sensitivity Function

**Figure 4:**
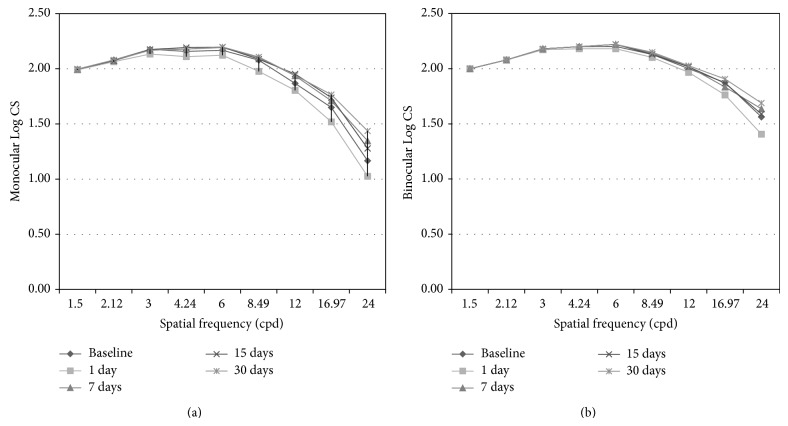
Monocular (a) and binocular (b) log contrast sensitivity (CS). Bars represent the Standard Error of the Mean (SEM) only for visits on days 1 and 30; the remaining error bars are omitted for clarity.

Monocular CSF results presented statistically significant changes over time for frequencies between 3.00 and 24.00 cpd (ANOVA, *P* < 0.05). Bonferroni post hoc correction showed that there was only a significant decrease in contrast sensitivity (CS) for frequencies 3.00 cpd to 8.49 cpd from baseline to day 1. Binocular CSF presented only statistically significant changes over time for 6.00 cpd (*P* = 0.024; ANOVA) and 24.00 cpd (*P* = 0.029; ANOVA) (Figure 4).

### 3.4. Correlations

Correlations between the outcome measures (light distortion, corneal higher order aberrations, and CSF) were obtained after the first night of lens wear, after 7 days, after 15 days, and after 30 days. It was observed that spherical-like and coma-like aberrations were inversely correlated with the irregularity of the light distortion (BFCirr) and the monocular CSF, respectively, after first night. These correlations were statistically significant after the first night of treatment for coma-like aberration with 3 mm pupil (*r* = −0.414 to *r* = −0.500; *P* < 0.001 for 12 and 24 cpd, resp.). Total RMS showed the higher and statistically significant correlation with CSF for 4.24 and 6 cpd after 7 days of treatment for the 3 mm pupil size (*r* = −0.529 and *r* = −0.551; *P* < 0.001). The correlations were weaker (*r* < 0.472) after first night and even lower after 1 month of treatment (*r* < 0.323).

At the 7 days' follow-up visit, light distortion parameters and corneal higher order aberrations were inversely correlated with monocular CS. Size (LDI and BFC_Rad_) and irregularity (BFC_Irreg_) were significantly correlated (*r* ≤ −0.400; *P* ≤ 0.013). These correlations were even higher at the 15 days' visit for LDI and BFCIrr with CS for 6 cpd spatial frequency (*r* ≤ −0.600; *P* < 0.001). Coma-like aberrations for 4.5 mm pupil size were also negatively correlated with CS for 4.24 and 6 cpd special frequencies (*r* ≤ −0.550; *P* < 0.001). Finally, after 1 month, the correlations between light distortion and corneal higher order aberrations with monocular CS were smaller (*r* ≥ −0.300; *P* ≥ 0.022) or absent.

## 4. Discussion

In the present study, BCVA was maintained at baseline level over the follow-up period. Ocular higher order aberrations significantly increased at 1 month after the procedure and remained stable thereafter. There was an initial loss in CS after overnight OK, and the loss persisted during the 1st-year follow-up. As a whole, all these parameters were stable throughout the posttreatment period from 1 month to 12 months. That is, posttreatment clinical parameters including refraction, visual acuity, corneal higher order aberrations, and CS were stable in the eyes that underwent overnight OK [7].

CS is a fundamental feature of vision, and its measurement provides useful information about visual function that may not be obtained by standard visual acuity testing [13]. CS has been subject of research in other fields such as refractive surgery, showing a significant decrease soon after the procedure [14, 15]. In the short term after both OK and corneal refractive surgery, corneal higher order aberrations increase and CSF decreases as the first response to treatment. In the long term, it has been reported that, in the case of refractive surgery, it recovers between 3 and 12 months after treatment, reaching values similar to baseline [16, 17]. Similar results have been found after OK treatment in the present study with faster recovery. This might be related with the fact that in OK treatment no healing processes are involved and CS is recovered once the treatment is stabilized. A potential limitation of our study is that we are considering corneal aberrations instead of total aberrations. This might justify the weak correlations between visual function as measured with CSF or light distortion and aberrations.

The impact of OK on visual performance, however, has not been studied in detail. There are few long-term OK studies in which the variation of higher order aberrations and CSF are studied [11] and one short-term study [10] in which light distortion is measured to investigate the correlation between pre- and posttreatment parameters and glare scores in OK subjects who had used OK lenses for more than 3 months.

In the present longitudinal study, we investigated changes in perception of light distortion, changes in corneal higher order aberrations, and CSF as representative of visual quality in eyes undergoing overnight OK for one year.

The analysis of light distortion surrounding a light source has proved to be effective in determining the time-course of changes in visual quality from the subject's perspective and irrespective of the corneal higher order aberrations pattern observed. A previous study by Villa et al. used a similar methodology but based on a software platform and a computer screen which is more limited in the presentation of bright stimuli. In their study, they reported an increase in light disturbances after corneal refractive surgery [18]. Lorente-Velázquez et al. used a different methodology to evaluate the effect of OK on the intraocular retinal stray light over a period of 1 month using the C-Quant instrument, based on the compensation-comparison method to derive retinal stray light [19]. They observed that minor changes in the stray light were not correlated with other psychophysical parameters as the CSF. Furthermore, the authors reported an improvement in retinal stray light after 1 month compared to baseline. This suggests that stray light does not reflect the impact of aberrations or any optical defects other than changes in the transparency of tissues. Similarly, Cerviño et al. were not able to find any significant effect of LASIK surgery on stray light values obtained with the C-Quant [20], while the analysis of halo size with a software system showed significant changes, as others had previously found [18]. Again, this supports the assumption that the light distortion reported here is not related to stray light phenomena.

We observed an inverse correlation between coma-like aberrations and CSF for medium and higher spatial frequencies, which might be related to the relatively frequent decentration and irregularity of the treatment zone after the first night wearing CL. Our results showed no significant correlation between the different optical (corneal aberrations) and quality of vision (CSF and light distortion) parameters at the end of the month. The absence of significant correlations at this stage might be interpreted as a neural adaptation process that is independent of the optical quality of the cornea. A hypothetical component of this neural adaptation might even take place at the retinal level, with a change in the apodization mechanism of the photoreceptors such that they overcome the effects of deteriorated peripheral optics of the eye and take advantage of the relatively unchanged optical quality within the more central part of the pupil. In fact, our results agree with previous reports by our group [5] that showed that the increase in corneal aberrations is hardly significant for a pupil size of 3 mm or less, what might explain visual quality remaining within normal limits in the midterm despite the initial deterioration. In another study analyzing subjective responses versus light distortion perception (whether it increases, decreases, or remains the same), we found that after 1 year of treatment 42.1% reported a decrease and the remaining 57.9% reported that the subjective feeling of light distortion remained the same [8]. In the present study, light distortion analysis showed a transient increase followed by a reduction to baseline levels after 7 days of treatment. This is in agreement with the clinical observation of adaptation to the distortion effect observed in our previous study [8]. Similarly, McAlinden et al., using the Quality of Vision Questionnaire (QoV) [21], reported a transient worsening of the subjective perception of quality of vision that returned to baseline a few weeks after laser-assisted subepithelial keratectomy [22]. Again, the results of the QoV questionnaires after surgery showed a slower recovery which might be related with the healing processes occurring in the cornea, which are not present in the OK treatment.

Interestingly, we observed a decrease in the distortion parameters related to the irregularity of the light distortion. This might be explained by the spherical aberration induced that leads to a more uniform and more radially symmetric distortion pattern compared to baseline, which will result in a reduction of the irregularities in the form of spikes surrounding the light source. Regarding aberration changes in the anterior corneal surface, an average increase of 110% for 3.0 mm pupil to 233% for 6.0 mm pupil in the spherical-like RMS was observed. Changes in comatic aberration were less severe and more similar for different pupil sizes ranging from 108% to 150% for 3.0 and 6.0 mm pupil sizes. Secondary astigmatism showed a marked increase ranging from 62% to 195% for 3.0 and 6.0 mm pupil sizes. Similar values have been reported by other studies [5, 6, 23].

To our knowledge, this is the first study addressing the short-term changes in the optical quality of the anterior corneal surface and the visual quality determined by means of monocular and binocular CSF and light distortion measurement. Although the light distortion and CSF recovered to baseline values, the lack of correlation after 15 days between light distortion and CSF might confirm that both methods are measuring different aspects of visual quality.

In summary, we have shown that despite the reduction in optical quality of the anterior corneal surface, some of the parameters related to visual quality of the eye return to being within normal range values within a narrow period of time, and this might be related with a neural adaptation process.

## Figures and Tables

**Table 1:** Demographic, refractive, and keratometric data of subjects (mean ± SD) and range (minimum and maximum).

Age	22.34 ± 8.08 years (18–43)
Sample (male/female ratio)	29 subjects (6/23)
M (baseline)	−2.10 ± 0.93 D (−1.00 to −4.75)
J0 (baseline)	−0.03 ± 0.14 D (−0.50 to 0.46)
J45 (baseline)	0.00 ± 0.08 D (−0.29 to 0.36)
Flattest keratometric radius	7.78 ± 0.28 mm (7.20 to 8.64)
Steepest keratometric radius	7.55 ± 0.29 mm (6.92 to 8.52)
Decimal VA (monocular)	1.16 ± 0.09 (0.90 to 1.50)

## References

[1] Lu F., Simpson T., Sorbara L., Fonn D. (2007). Corneal refractive therapy with different lens materials, part 2: effect of oxygen transmissibility on corneal shape and optical characteristics. *Optometry & Vision Science*.

[2] Queirós A., González-Méijome J. M., Villa-Collar C., Gutiérrez A. R., Jorge J. (2010). Local steepening in peripheral corneal curvature after corneal refractive therapy and LASIK. *Optometry and Vision Science*.

[3] Haque S., Fonn D., Simpson T., Jones L. (2004). Corneal and epithelial thickness changes after 4 weeks of overnight corneal refractive therapy lens wear, measured with optical coherence tomography. *Eye and Contact Lens*.

[4] Haque S., Fonn D., Simpson T., Jones L. (2007). Corneal refractive therapy with different lens materials, Part 1: corneal, stromal, and epithelial thickness changes. *Optometry and Vision Science*.

[5] Hiraoka T., Matsumoto Y., Okamoto F. (2005). Corneal higher-order aberrations induced by overnight orthokeratology. *American Journal of Ophthalmology*.

[6] Queirós A., Villa-Collar C., González-Méijome J. M., Jorge J., Gutiérrez A. R. (2010). Effect of pupil size on corneal aberrations before and after standard laser in situ keratomileusis, custom laser in situ keratomileusis, and corneal refractive therapy. *The American Journal of Ophthalmology*.

[7] Lu F., Simpson T., Sorbara L., Fonn D. (2007). The relationship between the treatment zone diameter and visual, optical and subjective performance in Corneal Refractive Therapy lens wearers. *Ophthalmic and Physiological Optics*.

[8] Santolaria E., Cerviño A., Queirós A., Brautaset R., González-Méijome J. M. (2013). Subjective satisfaction in long-term orthokeratology patients. *Eye and Contact Lens*.

[9] Queirós A., Villa-Collar C., Gutiérrez A. R., Jorge J., González-Méijome J. M. (2012). Quality of life of myopic subjects with different methods of visual correction using the NEI RQL-42 questionnaire. *Eye and Contact Lens*.

[10] Kojima T., Hasegawa A., Hara S. (2011). Quantitative evaluation of night vision and correlation of refractive and topographical parameters with glare after orthokeratology. *Graefe's Archive for Clinical and Experimental Ophthalmology*.

[11] Hiraoka T., Okamoto C., Ishii Y., Kakita T., Okamoto F., Oshika T. (2008). Time course of changes in ocular higher-order aberrations and contrast sensitivity after overnight orthokeratology. *Investigative Ophthalmology and Visual Science*.

[12] Linhares J. M. M., Neves H., Lopes-Ferreira D., Faria-Ribeiro M., Peixoto-de-Matos S. C., Gonzalez-Meijome J. M. (2013). Radiometric characterization of a novel LED array system for visual assessment. *Journal of Modern Optics*.

[13] Pesudovs K., Hazel C. A., Doran R. M. L., Elliott D. B. (2004). The usefulness of Vistech and FACT contrast sensitivity charts for cataract and refractive surgery outcomes research. *British Journal of Ophthalmology*.

[14] Holladay J. T., Dudeja D. R., Chang J. (1999). Functional vision and corneal changes after laser in situ keratomileusis determined by contrast sensitivity, glare testing, and corneal topography. *Journal of Cataract and Refractive Surgery*.

[15] Yamane N., Miyata K., Samejima T. (2004). Ocular higher-order aberrations and contrast sensitivity after conventional laser in situ keratomileusis. *Investigative Ophthalmology and Visual Science*.

[16] Mutyala S., McDonald M. B., Scheinblum K. A., Ostrick M. D., Brint S. F., Thompson H. (2000). Contrast sensitivity evaluation after laser in situ keratomileusis. *Ophthalmology*.

[17] Chan J. W. W., Edwards M. H., Woo G. C., Woo V. C. P. (2002). Contrast sensitivity after laser in situ keratomileusis one-year follow-up. *Journal of Cataract and Refractive Surgery*.

[18] Villa C., Gutiérrez R., Jiménez J. R., González-Méijome J. M. (2007). Night vision disturbances after successful LASIK surgery. *British Journal of Ophthalmology*.

[19] Lorente-Velázquez A., Nieto-Bona A., Collar C. V., Mesa A. G. (2011). Straylight and contrast sensitivity after corneal refractive therapy. *Optometry and Vision Science*.

[20] Cerviño A., Villa-Collar C., Gonzalez-Meijome J. M., Ferrer-Blasco T., García-Lázaro S. (2011). Retinal straylight and light distortion phenomena in normal and post-LASIK eyes. *Graefe's Archive for Clinical and Experimental Ophthalmology*.

[21] McAlinden C., Pesudovs K., Moore J. E. (2010). The development of an instrument to measure quality of vision: the quality of vision (QoV) questionnaire. *Investigative Ophthalmology and Visual Science*.

[22] McAlinden C., Skiadaresi E., Moore J. E. (2011). Visual and refractive outcomes following myopic laser-assisted subepithelial keratectomy with a flying-spot excimer laser. *Journal of Cataract and Refractive Surgery*.

[23] Hiraoka T., Okamoto C., Ishii Y., Kakita T., Oshika T. (2007). Contrast sensitivity function and ocular higher-order aberrations following overnight orthokeratology. *Investigative Ophthalmology and Visual Science*.

